# Fine Mapping of the *NRG1* Hirschsprung's Disease Locus

**DOI:** 10.1371/journal.pone.0016181

**Published:** 2011-01-20

**Authors:** Clara Sze-Man Tang, Wai-Kiu Tang, Man-Ting So, Xiao-Ping Miao, Brian Man-Chun Leung, Benjamin Hon-Kei Yip, Thomas Yuk-Yu Leon, Elly Sau-Wai Ngan, Vincent Chi-Hang Lui, Yan Chen, Ivy Hau-Yee Chan, Patrick Ho-Yu Chung, Xue-Lai Liu, Xuan-Zhao Wu, Kenneth Kak-Yuen Wong, Pak-Chung Sham, Stacey S. Cherny, Paul Kwong-Hang Tam, Maria-Mercè Garcia-Barceló

**Affiliations:** 1 Department of Psychiatry, University of Hong Kong, Hong Kong, China; 2 Department of Surgery, University of Hong Kong, Hong Kong, China; 3 Genome Research Centre, University of Hong Kong, Hong Kong, China; 4 Centre for Reproduction, Development and Growth, University of Hong Kong, Hong Kong, China; 5 Department of Surgery, Guiyang Medical College Affiliated Hospital, Guiyang, China; 6 Department of Epidemiology and Biostatistics, Ministry of Education Key Lab of Environment and Health, School of Public Health, Tongji Medical College of Huazhong University of Science and Technology, Wuhan, China; Innsbruck Medical University, Austria

## Abstract

The primary pathology of Hirschsprung's disease (HSCR, colon aganglionosis) is the absence of ganglia in variable lengths of the hindgut, resulting in functional obstruction. HSCR is attributed to a failure of migration of the enteric ganglion precursors along the developing gut. *RET* is a key regulator of the development of the enteric nervous system (ENS) and the major HSCR-causing gene. Yet the reduced penetrance of *RET* DNA HSCR-associated variants together with the phenotypic variability suggest the involvement of additional genes in the disease. Through a genome-wide association study, we uncovered a ∼350 kb HSCR-associated region encompassing part of the neuregulin-1 gene (*NRG1*). To identify the causal *NRG1* variants contributing to HSCR, we genotyped 243 SNPs variants on 343 ethnic Chinese HSCR patients and 359 controls. Genotype analysis coupled with imputation narrowed down the HSCR-associated region to 21 kb, with four of the most associated SNPs (rs10088313, rs10094655, rs4624987, and rs3884552) mapping to the *NRG1* promoter. We investigated whether there was correlation between the genotype at the rs10088313 locus and the amount of *NRG1* expressed in human gut tissues (40 patients and 21 controls) and found differences in expression as a function of genotype. We also found significant differences in *NRG1* expression levels between diseased and control individuals bearing the same rs10088313 risk genotype. This indicates that the effects of *NRG1* common variants are likely to depend on other alleles or epigenetic factors present in the patients and would account for the variability in the genetic predisposition to HSCR.

## Introduction

Hirschsprung's disease (HSCR, aganglionic megacolon) is a congenital disorder of the enteric nervous system (ENS) characterised by the absence of enteric ganglia along a variable length of the intestine. There is significant ethnic variation in the incidence of the disease, and it is most often found among Asians (2.8 per 10,000 live births)[1], [2]. Non-familial HSCR has a complex pattern of inheritance and manifests with low, sex-dependent penetrance and variability in the length of the aganglionic segment, according to which patients are classified into short segment (S-HSCR; 80%), long segment (L-HSCR; 15%), and total colonic aganglionosis (TCA; 5%). The male:female ratio is ≈4∶1 among S-HSCR patients and ≈1∶1 among L-HSCR patients. The recurrence risk to sibs of S-HSCR probands ranges between 1.5% and 3.3%, while risk to sibs of L-HSCR probands varies from 2.9% to 17.6%[1].

The *RET* gene, encoding a tyrosine-kinase receptor, is the major HSCR causing gene[3], [4] and its expression is crucial for the development of the enteric ganglia. Mutations in the coding sequence (CDS) of *RET* account for up to 50% of the familial cases and between 15%–20% of the sporadic cases[5]. Other HSCR genes identified so far mainly code for protein members of interrelated signalling pathways involved in the development of enteric ganglia: RET, endothelin receptor B (EDNRB), and the transcriptional regulator SOX10. Yet, mutations in genes other than *RET* account for only 7% of the cases[6], [7], [8], [9], [10], [11], [12], [13]. Despite the importance of *RET*, additional genes (acting either in conjunction with or independently from *RET*) are necessary to explain not only the disease incidence but also its complex pattern of inheritance.

Through a genome-wide association study (GWAS) on Chinese individuals we identified the association of a 350 kb genomic region encompassing intron 1 of the *NRG1* gene with Hirschsprung's disease[14]. Within the *NRG1* region, the strongest overall associations were found for two SNPs located in intron 1 of the neuregulin1 gene (*NRG1*) on 8p12, with rs16879552 and rs7835688 (underlined throughout the text; supplementary Figure S1) yielding odds ratios of 1.68 [CI95%:(1.40,2.00), *p* = 1.80×10^−8^] and 1.98 [CI95%:(1.59,2.47), *p* = 1.12×10^−9^], respectively, for the heterozygous risk genotypes under an additive model. *NRG1* plays an important role in ENS development and maintenance[15], [16], [17], [18].

As these intron 1 *NRG1* HSCR-associated SNPs are not predicted to functionally affect the gene, we hypothesized that these loci are in linkage disequilibrium (LD) with a functional variant(s) not covered by the 500K *Affymetrix* chips used in our initial GWAS. To find those functional variant(s), we increased the genotype density within the region by genotyping 243 SNPs in 343 HSCR Chinese HSCR patients and 380 Chinese controls. Genotype imputation was used to further increase the SNPs density. As the *NRG1* HSCR-associated region encompasses regulatory regions, we hypothesized that *NRG1* SNPs could affect HSCR susceptibility by altering *NRG1* expression. Thus, the difference between *NRG1* expression levels in gut tissues of affected and non-affected individuals was tested, along with the relationship of expression with *NRG1* genotype.

## Results and Discussion

### Fine mapping implicates the NRG1 promoter in HSCR susceptibility

Fine mapping of *NRG1* was carried out by genotyping additional SNPs within the region delimited by the upstream and downstream recombination hot spots. We found 9 SNPs (“typed” in Table 1) more significantly associated with HSCR than rs7835688 (initially discovered in the GWAS; *p* = 5.92×10^−4^; OR = 1.73 and 95% CI = 1.28–2.32 in this set) and one SNP with a *p*-value lower than that of the also GWAS identified rs16879552 (*p* = 8.99×10^−5^; OR = 1.63 and 95% CI = 1.29–2.07; also in this set).

**Table 1:** Fine mapping association results of *NRG1* SNPs using logistic regression on MACH-imputed allelic dosage

		MAFa, ,b		Allele^c^			Before imputation	Association values after imputation		
SNP	Position	Cases	Controls	Minor	Major	Type	*P*	OR	(95%CI)	*P*
rs16879425	32426748	0.44	0.34	A	C	typed	2.29E-04	1.57	(1.23,1.99)	2.21E-04
rs10954845	32439384	0.35	0.25	A	G	typed	2.57E-04	1.59	(1.24,2.05)	3.29E-04
rs4422736	32490062	0.45	0.35	C	T	typed	4.00E-04	1.54	(1.20,1.96)	4.00E-04
rs10113578	32503670	0.51	0.40	G	A	imputed	NA	1.53	(1.22,1.94)	3.18E-04
**rs10088313**	**32509603**	**0.52**	**0.40**	**G**	**T**	**typed**	**6.71E-05**	**1.60**	**(1.27,2.02)**	**6.71E-05**
rs10107065	32510100	0.52	0.40	A	G	imputed	NA	1.60	(1.27,2.01)	7.65E-05
rs10113593	32510900	0.51	0.40	T	C	typed	2.18-04	1.55	(1.23,1.96)	2.04E-04
rs10094655	32513689	0.52	0.39	T	A	imputed	NA	1.63	(1.29,2.05)	4.27E-05
rs4624987	32516246	0.52	0.40	G	A	imputed	NA	1.62	(1.29,2.05)	4.69E-05
rs3884552	32519399	0.53	0.42	C	T	imputed	NA	1.65	(1.30,2.09)	4.13E-05
rs7826312	32519657	0.24	0.14	C	T	typed	3.22E-04	1.77	(1.30,2.41)	3.22E-04
rs3802159	32524243	0.51	0.39	G	C	typed	1.25E-04	NA	NA	NA
rs7834206	32525690	0.27	0.16	A	C	typed	2.27E-04	NA	NA	NA
rs16879552	32530758	0.51	0.38	C	T	typed	8.99E-05	1.63	(1.29,2.07)	4.19E-05
rs7835688	32531041	0.26	0.15	C	G	typed	5.92E-04	1.73	(1.28,2.32)	3.30E-04
rs16879576	32560777	0.51	0.41	C	A	imputed	NA	1.57	(1.24,1.99)	1.87E-04
rs12680129	32562687	0.51	0.41	A	G	typed	1.86E-04	1.57	(1.24,1.98)	1.83E-04

Only SNPs with *P*-value lower than either or both of the 2 previously implicated SNPs (rs16879552 and rs7835688) are shown;

a : minor allele frequency; underlined: SNPs found associated in the previously reported GWAS.

b : frequencies reported for imputed alleles (except for rs3802159 and rs7834206);

c : minor and major alleles in patients and controls combined; in bold genotyped SNP with the lowest *p* association value.

**Figure 1:**
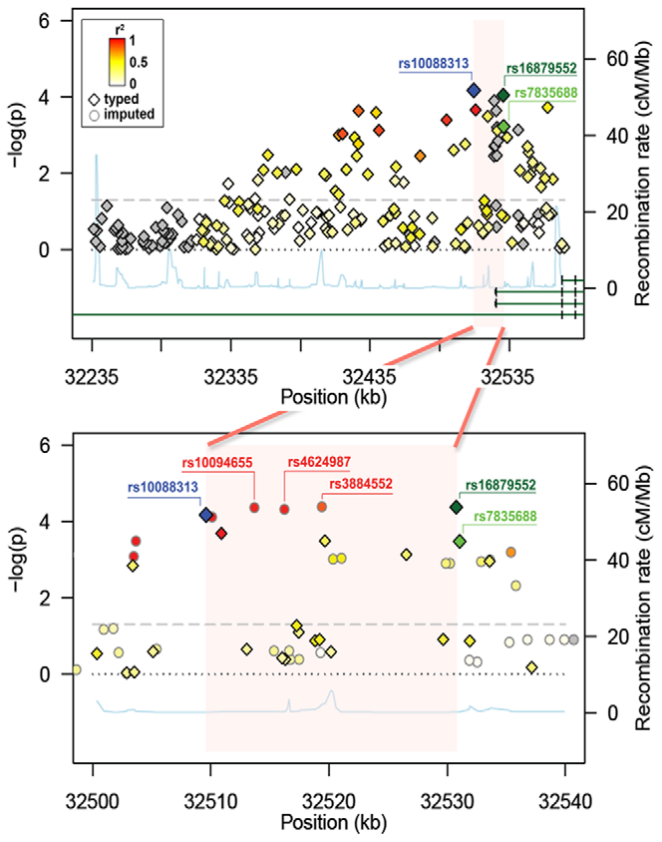
Fine mapping association results of NRG1 SNPs. Association results shown before (upper panel) and after imputation (lower panel). Diamonds and circles indicate genotyped and imputed SNPs respectively. Colour gradient (red r^2^ = 1 to white r^2^ = 0) marks the LD of the SNPs with rs10088313, except green for rs7835688, dark green for rs16879552 and blue for itself; grey indicates no information on LD. The 21 kb region we narrowed down to is highlighted in pink. The fine-scale recombination rate across the region is represented by the light-blue line. Green lines symbolize a schematic representation of the *NRG1* isoforms overlapping the associated region. The bottom green line represents the GGF2 isoform associated with schizophrenia that expands 900 kb upstream the transcription start site for the rest of *NRG1* isoforms.

The SNP displaying the strongest association (rs10088313; in bold face in Table 1) mapped to the promoter region of most *NRG1* isoforms, (except GGF2, associated with Schizophrenia[19]), and had the strongest LD (r^2^ = 0.84) with rs16879552. Other top SNPs were also in moderate to high LD with the previously GWAS-implicated SNPs (rs7835688 and rs16879552) (Figure 1, upper panel).

We next tried to refine our findings by imputing un-typed SNP allelic dosage using MACH. Imputation (based on HapMap Phase II haplotypes) nominated three additional HSCR-associated SNPs, rs10094655, rs4624987 and rs3884552, with highly similar level of association as rs16879552. In particular, rs3884552 displayed the highest significance level among all (Table 1). These markers fall again into the promoter of *NRG1*, about 10 kb upstream of rs16879552. Together with rs10088313, all five SNPs (rs10088313, rs10094655, rs4624987, rs3884552, and rs16879552) are highly correlated and have a similar MAF in both cases and controls. Beyond the region set by rs10088313 and rs16879552, we did not found any SNPs of similar levels of LD and significance. Thus, fine mapping coupled with imputation identified 5 highly associated SNPs (highlighted in grey in Table 1) narrowing-down the *NRG1* associated region from 350 kb to 21.155 kb (Figure 1, lower panel). Haplotype analysis did not result in any haplotype appreciably more associated with HSCR than any single SNP.

As further genotyping and statistical genetic analysis of these 5 equally associated SNPs in an expanded sample would not help discern causality among these linked SNPs, we resorted to comparative genomics for the identification of conserved regulatory regions overlapping/neighbouring these loci as this would point at the functional SNP. Two conserved regions overlapping the two associated SNPs, rs10088313 and rs4624987 respectively, were observed (see Supplementary Figure S2). In particular, the region encompassing rs4624987 associates with signals of H3K4me1 and H3K27Ac chromatin activity, suggesting the presence of an enhancer. Importantly, rs4624987 falls in the vicinity of a transcription factor binding site (TFBS) for the transcription factor (TF) EVI1 (PhastCon 28-way score >0.93). No other described regulatory elements were identified. Even though the TFBS for EVI1 does not overlapped with rs4624987, the SNP associated allele might introduce a new site or reduce access to the predicted site located only a few base-pairs away. According to the literature reviewed, mice homozygous for targeted null *Evi1* mutations are embryonic lethal and are characterized by widespread hypocellularity and poor/disrupted development of the cardiovascular and neural crest-derived cells[20].

As current data do not allow us to single out any of the four SNPs, we would argue that, most likely, they collectively represent a single signal of association and the lesser associated SNPs in the region are the aftermath of the indirect association. The fine-mapping of this *NRG1* region may not been effective due to the high correlation among SNPs. Yet, the 350 kb region has been narrowed-down to a 21 kb region within which the most HSCR-associated *NRG1* SNPs point at the *NRG1* promoter as the culprit. Plausibly, the HSCR-associated alleles are bound to alter the regulation of *NRG1* transcription and confer susceptibility to HSCR by affecting, in turn, the role of NRG1 signalling during ENS development.

#### HSCR-associated SNPs and NRG1 expression levels in human gut

If the *NRG1* HSCR-associated SNPs identified in the promoter are functional (or in LD with a causal variant not discerned due to LD), they are likely to affect the gene expression. Thus, we next investigated whether there was a correlation between the genotypes of the *NRG1* HSCR-associated SNPs and the levels of expression for this gene in human gut. Even though the causal variant cannot be pinpointed due to the high LD in the narrowed fine-mapped region, we assumed that the genotypes of the typed rs10088313 would represent those of the causal variant.

Due to alternative splicing, the *NRG1* gene generates at least 3 main types (Type I, II and III) of proteins and at least 31 isoforms[19]. As each *NRG1* type may have a different tissue-specific function, we assessed by RT-PCR which of the three main *NRG1* types[15] was mainly expressed in human gut (data not shown). We detected expression of *NRG1* Type I and vestigial expression of *NRG1* type III (**S**ensory and **M**otor-neuron **D**erived **F**actor; SMDF; NM_013959.2). We did not detect expression of NRG1 type II. The feeble expression of *NRG1* Type III detected in some samples may be due to the peripheral nervous system innervations of the gut, which is independent of the ENS.

In order to assess the effect of the *NRG1* HSCR-associated SNPs on the expression of *NRG1* type I in human gut, we used quantitative real-time PCR. We would also like to point out that as *NRG1* is expressed in both intestinal mucosa and enteric ganglia[14], we did not discriminate between the different gut cellular types and assumed that the effect of the *NRG1* regulatory SNPs would indiscriminately affect the total amount of *NRG1* expressed in the gut.

**Figure 2:**
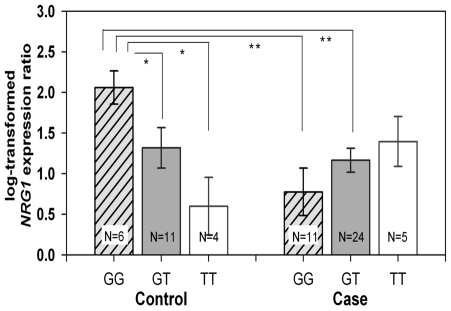
NRG1 expression in human gut tissues. Quantitative RT-PCR analysis of log-transformed expression ratio of *NRG1* to 18S in the colon tissues from the normal portions of HSCR patients and non-HSCR patients stratified according to the rs10088313 (G/T; G risk allele). Bars represent the standard error (SE). * *p*<0.05; ** *p*<0.005.

The overall *NRG1* expression in gut did not differ between patients and controls (F = 2.18, *p* = 0.15, two way ANOVA) as well as among genotypes for the combined samples (F = 0.29, *p* = 0.75). Significant interaction between genotype and case-control status (F = 5.54, *p* = 0.0064) was found, indicating that the relationship between genotype and expression level differs between cases and controls. When both groups (cases and controls) were stratified according to the rs10088313 genotypes (**GG**, **G**T, TT; risk allele in bold), *post hoc* pair-wise t-tests resulted in statistically significant differences among the three genotypes only within the control group (Figure 2). In this group, the highest *NRG1* expression levels were observed in individuals with the GG (risk) genotype and the lowest in individuals with the TT genotype. Furthermore, GG in controls shows higher expression than GG in cases. These data suggest that the *NRG1* risk allele has opposite effects on patients compared to controls. This may be explained by an additional susceptibility factor/s (mainly present in the HSCR group) interfering with the role played by rs10088313 in *NRG1* regulation. This allows us to hypothesize the presence of *trans*-acting elements that interact with *NRG1* regulatory sites modulating their genetic effect on *NRG1* transcription. In this context, patients' specific mutations or polymorphisms in the gene encoding the predicted EVI-1 TF could account for the different effects observed for the rs10088313 G allele. Another attractive hypothesis would be that the effect of the rs10088313 G allele was dependant on the *RET* risk alleles. This would be backed-up by our finding of genetic interaction between the *RET* rs2435357 (C/T) T risk allele and the *NRG1* risk alleles identified in our GWAS[14], [21]. Importantly, while most of the controls with rs10088313 GG genotypes (N = 6) did not harbour the *RET* risk allele T (5 were CC and 1 CT for rs2435357), most patients with rs10088313 GG genotype (N = 11) had the *RET* risk allele T (7 were TT and 2 CT for rs2435357). We previously showed that this *RET* rs2435357T HSCR risk allele is associated with reduction of *RET* gene expression in gut tissues[22]. Thus, stratified analysis of *NRG1* expression according to the *RET* rs2435357 genotypes would have helped elucidate if the reduction in *RET* expression relates to that of *NRG1* and could perhaps account for the differences encountered in *NRG1* expression between patients and controls with the same *NRG1* genotype. We could not perform such stratified analysis as the sample size is not large enough and because the high frequency of the *RET* risk in the Chinese population prevents us from obtaining a large enough representation of all *RET* genotypes.

We have recently demonstrated that the *RET* rs2435357 T risk allele disrupts a SOX10 binding site that compromises *RET* transactivation[23] SOX10 is also known to regulate the expression of NRG1 receptor ErbB3 in neural crest cell receptors[15] Though the link between RET and NRG1 signalling in the development of the ENS is still to be elucidated our data suggests that NRG1 is player in the signaling network implicated in ENS development and maintenance and that may genetically and biologically interact with members of the RET signalling pathway during the ENS development. Biological interaction between RET and NRG1 signaling has been reported and linked to the survival and maintenance of the peripheral nervous system, where injury-induced expression of the RET ligand GDNF by non-myelinating Schwann cells is ErbB dependent[24].

We suspect that our expression data may be reflecting the result of the genetic *NRG1*-*RET* interaction previously described[14].

Somehow comparable findings have been reported in a study on *NRG1* expression in post-mortem human brain samples of schizophrenic patients and control individuals where *NRG1* expression levels differ between patients and controls for a given *NRG1* schizophrenia susceptibility SNP genotype[25]. Collectively, these findings warrant further study on *NRG1* regulation and its implication in diseases.

This expression study has limitations, some of which cannot be overcome. Firstly, the gut of newborn patients is in an advanced developmental stage (although not fully mature). Thus our analysis does not mimic the expression of *NRG1* during the early developmental stages of the human gut, when expression patterns of other genes may be different. For obvious reasons, this cannot be surmounted. Secondly, also for obvious reasons, it is not possible to obtain gut tissue samples from controls, requiring us to use samples from individuals who underwent gut biopsy for other motives than HSCR. Thirdly, the limited sample size is hampering the expression study. Again, it is difficult to obtain a balanced representation of all *NRG1* SNP genotypes and a desirable number of gut tissues from affected and non-affected individuals.

We conclude that *NRG1* regulatory SNPs may confer an increased risk of HSCR by interfering with the normal *NRG1* expression in human developing gut and their effect is likely to depend on the genetic background of the individual, most likely DNA alterations on *trans*-acting regulatory proteins expressed in the developing gut in a time-specific manner or in other main HSCR genes such as *RET*. Why and how altered *NRG1* expression may contribute to HSCR is yet to be learned.

## Materials and Methods

### Ethics Statement

At recruitment, informed consent was obtained from each subject. This study was approved by the institutional review board of the University of Hong Kong (UW 03-227 T/227).

### Subjects

A total 343 ethnic Chinese patients diagnosed with sporadic HSCR were included for genotyping. Of those, 258 HSCR had been included in our previous GWAS[14] and 177 in the fine mapping of the 9q31 HSCR susceptibility locus[26]. The characteristics of the patients are summarized in supplementary Table S1.

Control individuals were obtained from the blood bank of the Hong Kong Red Cross. We included a total of 359 ethnic Chinese subjects without a diagnosis of HSCR.

Gut tissues were collected from 40 HSCR patients (subset of the 343 individuals genotyped) and 21 non-HSCR patients who had undergone colon surgery for reasons other than HSCR. For the 21 non-HSCR patients (12 affected with imperforate anus; 7 with necrotizing enterocolitis and 2 with mesenteric cysts), tissues were obtained from at least 2 cm away from the margin of the diseased bowel.

The HSCR diagnosis was histologically confirmed with either biopsy or surgical resection material for absence of enteric plexuses.

#### SNP selection

Following the identification of the *NRG1* rs16879552 and rs7835688 HSCR-associated SNPs and the delimitation of the 350 kb HSCR-associated region (chromosome 8:32.235–32.575 Mb; hg18) as originally described, we proceeded with fine-mapping.

The 350 kb region is flanked by two major recombination hotspots (A: 32.235–32.245 Mb and C:32.565–32.575 Mb). A close-up look at the recombination rate within the region (downloaded from HapMap Phase II database) revealed an additional hotspot (B: 32.395–32.405 Mb) that disrupts slightly the LD of the main haplotype block (Supplementary Figure S1). LD was found to be stronger in the region flanked by hotspots B and C. This B–C region spans from the 5′ UTR to exon 2 of the *NRG1* isoforms (except for isoform GGF-2 -**G**lial **G**rowth **F**actor-; NM_013962.2) and includes mostly non-coding sequence DNA containing conserved noncoding elements (CNEs) which are likely to control gene regulation, chromosome structure, and other key functions.

Annotation and sequences of the DNA polymorphisms in the 350 kb region were downloaded from the NCBI dbSNP database (dbSNP129). Among the 2675 polymorphisms downloaded, we excluded 520 multiallelic variants and those known to be monomorphic SNPs in Asians. SNPs with MAF as low as 1% in HapMap were not excluded as we attempted to fine map the casual variant affecting the disease, which could be quite rare in the general population.

In order to minimize the genotyping cost, we used WCLUSTAG[27], an in-house developed tagging program, to identify tag SNPs among the HapMap genotyped SNPs (r^2^>0.90).

For selection of SNPs without population frequencies available, we applied a tiered approach based on their functional significance: **i**) we force-included all nonsynonymous SNPs, since presumably they have higher impact on protein structure and function; and **ii**) we selected SNPs according to functional scores. For this, we relied on two databases, Ensembl and UCSC. The criteria for selection included accessibility to chromatin, CpG islands associated with promoter and the degree of conservation among species (Multiz 28-way). For each SNP, we also checked if the allelic variation introduces potential change in predicted transcription factor binding sites (TFBS). To this end, we used P-MATCH[28]. We assigned a higher score to SNPs whose variation disrupts an existing TFBS or creates a new one.

A total of 243 SNPs spanning about 350 kb of chromosome 8 (from 32.236 Mb to 32.575 Mb) were selected for genotyping.

#### SNP genotyping

The 243 SNPs selected were genotyped in 343 HSCR cases and in 359 controls using Sequenom technology as previously described[29]. After removing 12 cases and 8 controls with call rate <90%, 331 HSCR cases and 351 controls remained for association analysis. Standard quality control criteria for SNPs were employed, leaving a total of 207 SNPs with call rate >95%, MAF>1% and not violating Hardy Weinberg equilibrium (*p*>0.001).

#### Real-time assay for gene expression

Resected colon tissues were collected from 40 HSCR patients and 21 non-HSCR patients. No tissues were available from the rest of the patients. All resected tissues were immediately placed in liquid nitrogen and then stored at –80°C before analysis. Full-thickness tissues from ganglionic portions of bowel of each HSCR patient and colons from non-HSCR patients were used for RNA extraction by Trizol Reagent (Life Technologies, Rockville, MD) and converted to cDNA using an oligo (dT)15 primer and Superscript III (Invitrogen, Carlsbad, CA). The cDNA products equivalent to 10 ng of total RNA were used for quantitative real-time PCR which was performed by ready-to-use TaqMan gene expression assays from Applied Biosystems. The assay for *NRG1* was Hs00247620_m1, which targets all but type 3 *NRG1* SMDF (this isoform plays a major role in myelination). Real-time qPCR was performed in triplicate (96-well plates) on an ABI 7900 (Applied Biosystems) machine using standard thermal cycling conditions (10 min at 95°C, 40 cycles for 15 s at 95°C, 1 min at 6°C). A standard curve was constructed for each PCR run with 10-fold serial dilutions containing 100, 10, 1, 0.1 and 0.01 ng/mL of cDNA from the neuroblastoma cell line HTB11. The amount of target gene per sample was interpolated according to the standard curves. All analyses were performed in a blinded fashion with the laboratory operators unaware of genotyping data.

#### Statistical analysis

Association was assessed by means of R and PLINK[30] using logistic regression under an additive model and sample origin (Northern *vs*. Southern Chinese) was included as a covariate to correct for population stratification.

To further evaluate the association of untyped markers tagged by the genotyped SNPs, imputation was carried out by MACH[31] using HapMap Phase II CHB haplotypes as reference. As our ultimate goal here was to pinpoint the causal variant of *NRG1*, increasing marker density outweighed the use of the less dense yet more accurate Phase III panel.

We examined the effects of case-control status and *NRG1* genotype on log-transformed *NRG1* expression in gut via two-way ANOVA and post-hoc test of all pair-wise differences. These statistical analyses were done using R. All statistical tests were two-sided, and *p*<0.05 was considered significant.

## Supporting Information

Table S1 **Characteristics of the Chinese HSCR patients included in the NRG1 genotyping.**
(DOCX)

Figure S1 **Schematic representation of the 350 kb NRG1 HSCR-associated region.** On the top panel, close-up of the chromosome 8 association peak obtained in the GWAS. Middle panel, recombination rates throughout the region (red vertical lines). *NRG1* isoforms are represented by grey lines (boxes represent exons). Bottom line, Haploview representation of the LD in the region (D′).
(TIF)

Figure S2 **Regional map of the 5 HSCR-associated SNPs**. rs10088313, rs10094655, rs4624987, rs3884552 and rs16879552 depicted in top green panel, from left to right. Conservation information was given by PhastCon score for Multiz 28-way alignment for vertebrates (hg18).
(TIF)
