# Supplementary material for: Fine Mapping of the NRG1 Hirschsprung's Disease Locus
Source: PLoS One. 2011 Jan 20;6(1):e16181. doi: 10.1371/journal.pone.0016181 (PMC3024406; doi:10.1371/journal.pone.0016181)
Supplement: Table S1 — Characteristics of the Chinese HSCR patients included in the NRG1 genotyping. (DOCX) [file pone.0016181.s001.docx]

| **Supplementary Table S1: characteristics of the Chinese HSCR patients included in the *NRG1* genotyping** | | | | | | | | |
| --- | --- | --- | --- | --- | --- | --- | --- | --- |
| N=331^a^ | S-HSCR n=248 | | L-HSCR n=28 | | TCA n=14 | | Undetermined n=41 | |
|  | Males | Females | Males | Females | Males | Females | Males | Females |
|  | 200 {21} (10) | 48 {2} (3) | 17{1} | 11 {1} | 9 {2} | 5 | 32 | 9 {1} |
| ^a^number of patients after quality control filtering. { } additional anomalies; ( ) Down syndrome; U: undetermined | | | | | | | | |
