# Supplementary figures and images for: Fine Mapping of the NRG1 Hirschsprung's Disease Locus
Source: PLoS One. 2011 Jan 20;6(1):e16181. doi: 10.1371/journal.pone.0016181 (PMC3024406; doi:10.1371/journal.pone.0016181)

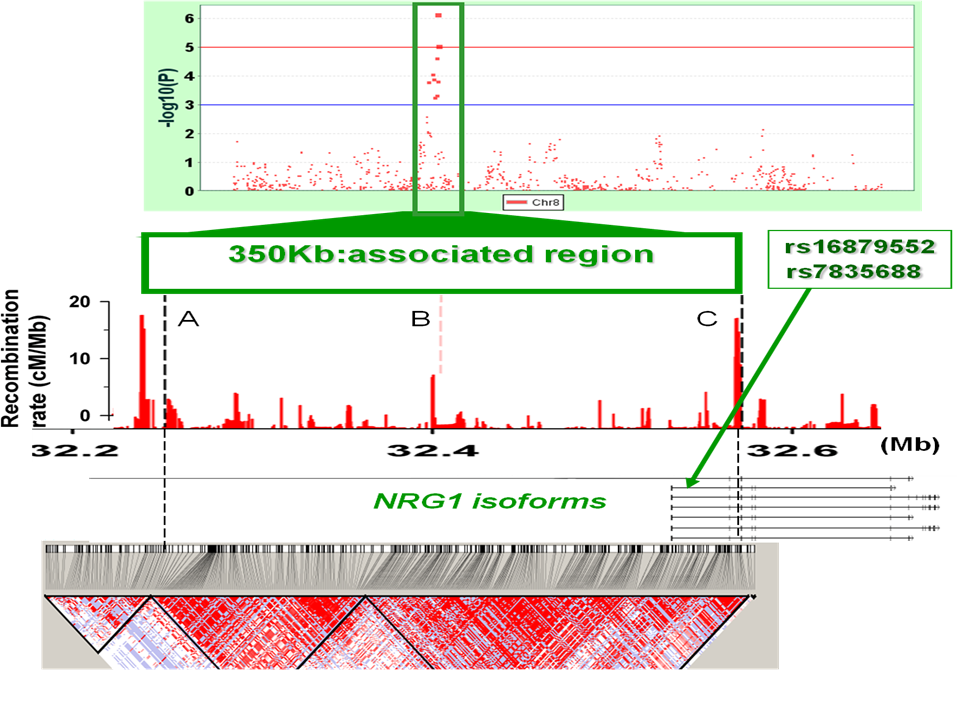

Supplement: Figure S1 — Schematic representation of the 350 kb NRG1 HSCR-associated region. On the top panel, close-up of the chromosome 8 association peak obtained in the GWAS. Middle panel, recombination rates throughout the region (red vertical lines). NRG1 isoforms are represented by grey lines (boxes represent exons). Bottom line, Haploview representation of the LD in the region (D′). (TIF) [file pone.0016181.s002.tif]

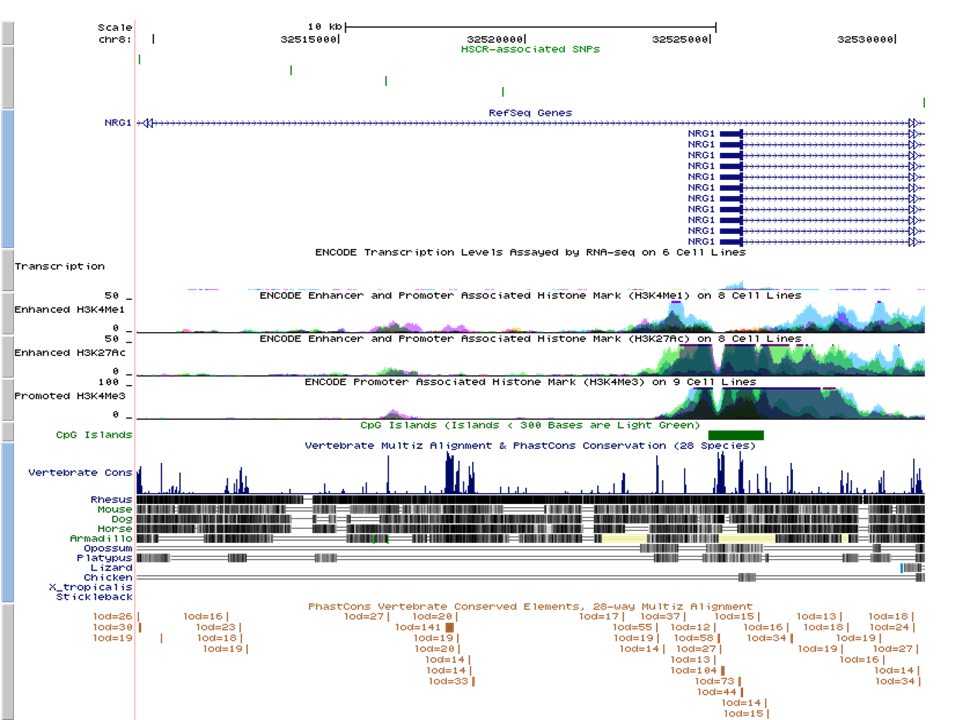

Supplement: Figure S2 — Regional map of the 5 HSCR-associated SNPs. rs10088313, rs10094655, rs4624987, rs3884552 and rs16879552 depicted in top green panel, from left to right. Conservation information was given by PhastCon score for Multiz 28-way alignment for vertebrates (hg18). (TIF) [file pone.0016181.s003.tif]
